# Making PI3K superfamily enzymes run faster

**DOI:** 10.1016/j.jbior.2024.101060

**Published:** 2024-11-19

**Authors:** Grace Q. Gong, Madhangopal Anandapadamanaban, Md Saiful Islam, Iain M. Hay, Maxime Bourguet, Saulė Špokaitė, Antoine N. Dessus, Yohei Ohashi, Olga Perisic, Roger L. Williams

**Affiliations:** a MRC Laboratory of Molecular Biology, Francis Crick Avenue, Cambridge, CB2 0QH, UK; b University College London Cancer Institute, University College London, London, UK

## Abstract

The phosphoinositide 3-kinase (PI3K) superfamily includes lipid kinases (PI3Ks and type III PI4Ks) and a group of PI3K-like Ser/Thr protein kinases (PIKKs: mTOR, ATM, ATR, DNA-PKcs, SMG1 and TRRAP) that have a conserved C-terminal kinase domain. A common feature of the superfamily is that they have very low basal activity that can be greatly increased by a range of regulatory factors. Activators reconfigure the active site, causing a subtle realignment of the N-lobe of the kinase domain relative to the C-lobe. This realignment brings the ATP-binding loop in the N-lobe closer to the catalytic residues in the C-lobe. In addition, a conserved C-lobe feature known as the PIKK regulatory domain (PRD) also can change conformation, and PI3K activators can alter an analogous PRD-like region. Recent structures have shown that diverse activating influences can trigger these conformational changes, and a helical region clamping onto the kinase domain transmits regulatory interactions to bring about the active site realignment for more efficient catalysis. A recent report of a small-molecule activator of PI3Kα for application in nerve regeneration suggests that flexibility of these regulatory elements might be exploited to develop specific activators of all PI3K superfamily members. These activators could have roles in wound healing, anti-stroke therapy and treating neurodegeneration. We review common structural features of the PI3K superfamily that may make them amenable to activation.

## 1 Introduction

**Fig. 1:**
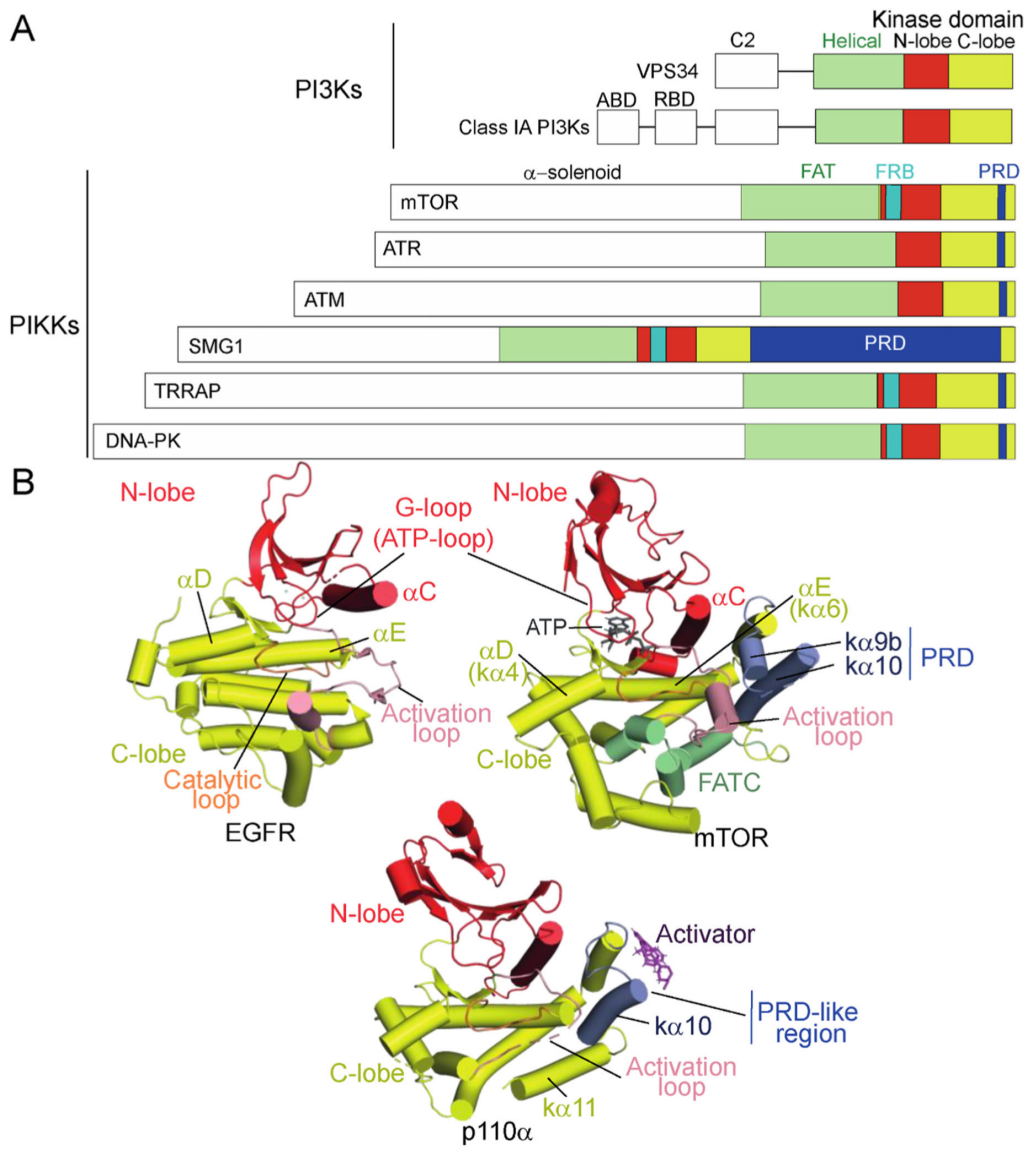
The PI3K superfamily of enzymes have a conserved kinase domain at the C-terminus. A. The domain organizations of the PI3Ks (upper) and PI3K-related protein kinases (PIKKs) (lower). B. The PI3K superfamily members have a kinase domain that belongs to the atypical protein kinases but share a common fold with the eukaryotic protein kinases (ePKs). The kinase domain from the EGFR ePK (left, PDB ID 5UGB) is illustrated next to the mTOR kinase domain (right, PDB ID 6BCX). The N-lobes of EGFR and mTOR were superimposed to get a common orientation, since the N-lobes have a more similar structure than the C-lobes. The β-sheet and helix αC of the N-lobe as well as helices αD and αE in the C-lobe have a similar arrangement in ePKs and the PI3K superfamily. The rest of the helices in the C-lobe of ePK have no structural homology to PI3K superfamily. A ribbon diagram of the kinase domain from the complex of p110α with the 1938 activator (PDB ID 8OW2) is shown below. The PI3K kinase domain has a structure that closely resembles the PIKKs, although the last helix of the kinase domain (kα12) has a very different structural and functional role in PIKKs and PI3Ks: in PIKKs, it is part of the FATC that plays a role in structural integrity of the C-lobe, while in PI3Ks it is flexibly attached (in most structures not visible) and is critical for membrane binding. A PRD-like structural feature (blue) is present both in the PI3Ks and the PIKKs, and this is typically an important element involved in regulation of the enzymes. The ePKs have no analogous element. For mTOR, the FRB helical insertion in the N-lobe was omitted for clarity.

**Fig. 2:**
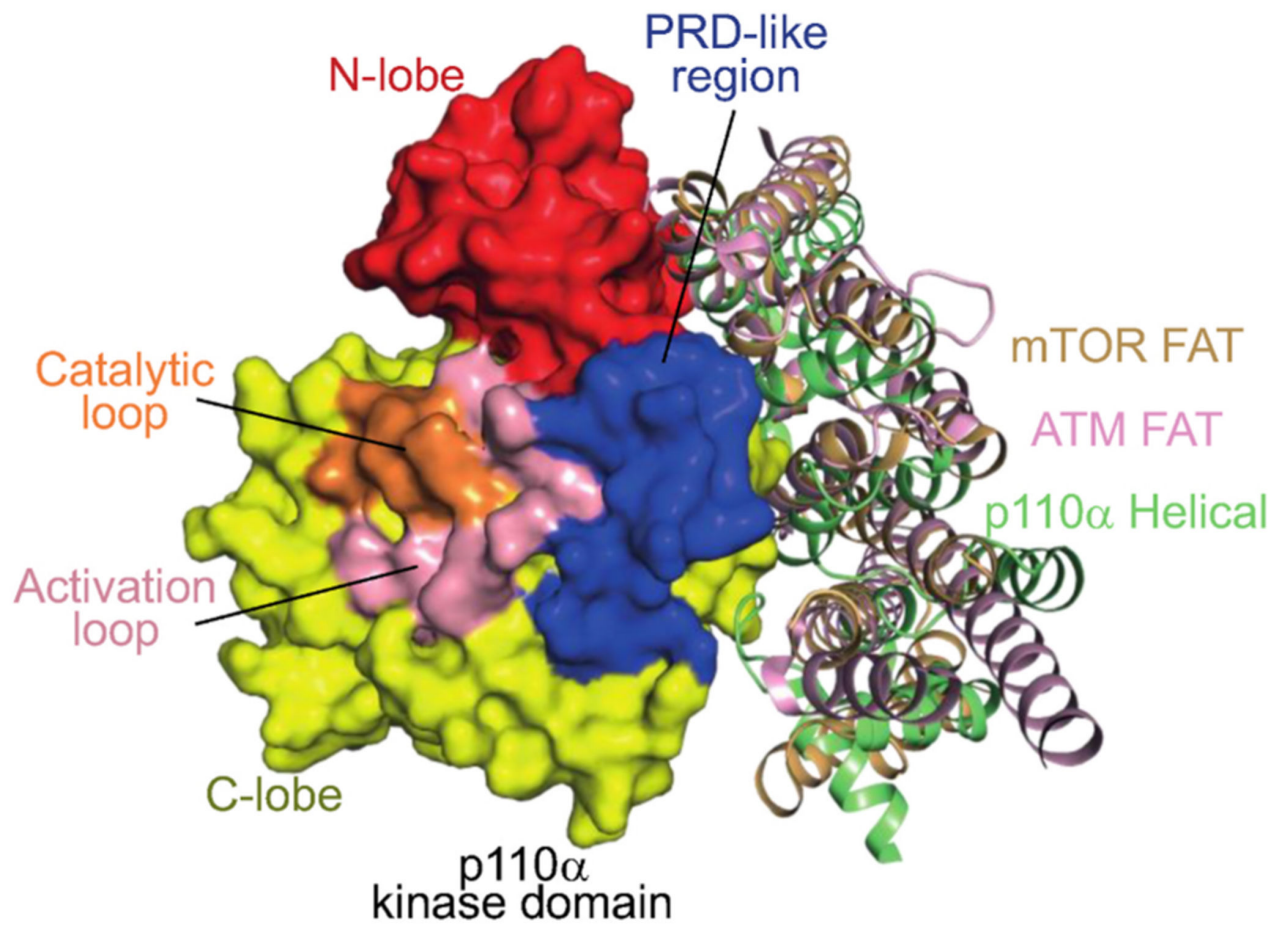
PIKKs and PI3Ks have helical solenoid elements that form tight associations with the kinase domains, and although they are not homologous, these helical regions are structurally similar. These elements are known as the FAT (PIKKs) and helical (PI3Ks) domains. The FAT domains are homologous among PIKKs, and the helical domains are homologous among PI3Ks. However, there is no sequence conservation between the FAT and helical domains. The helical/kinase unit is illustrated for p110α (green helical domain, PDB ID 8BFU) along with the C-terminal portion of the mTOR FAT (light orange, PDB ID 6BCX) and ATM FAT (pink, PDB ID 7SIC). The structures were superimposed on the C-lobes of the kinase domains.

Eukaryotes evolved the PI3K superfamily of closely related kinases that includes both lipid kinases (PI3Ks and type III PI4Ks) and PI3K-like Ser/Thr protein kinases (PIKKs). In mammalian cells, the PIKKs include protein kinases mTOR, DNA-PK, ATM, ATR, SMG1 and the pseudokinase TRRAP. The superfamily members have in common a bi-lobal kinase domain with a core that is only distantly related to the typical eukaryotic protein kinases (ePKs) (Kanev et al., 2019; Modi and Dunbrack, 2019). As such, they have been grouped into a heterogenous collection of kinases referred to as the atypical protein kinases. The recognizable kinase core shared by both ePKs and PI3K superfamily consists of two lobes, a smaller N-lobe and a larger C-lobe, with a deep active-site cleft between them that accommodates ATP/Mg^2+^ (Fig. 1). The conserved elements of the N-lobe include a five-stranded β-sheet associated with an ATP-phosphate-binding β-hairpin loop called the glycine-rich loop (or G-loop) in ePKs and a helix known as αC. In most members of the PI3K superfamily, the β hairpin analogous to the G-loop in ePKs does not have glycines. Therefore, here we will refer to this structural element as the ATP-loop, instead of the more intuitive name of phosphate binding loop (P-loop), to avoid any possible confusion with the unrelated Rossman fold P-loop (β-strand followed by a glycine-rich loop and a helix). The conserved elements in the C-lobe include two helices (αD and αE) followed by the catalytic loop, a small two-stranded β-sheet, and the activation loop (Kanev et al., 2019; Modi and Dunbrack, 2019). The conserved Asp from the catalytic loop interacts with the hydroxyl of the substrate acceptor (Fig. 1). In both ePKs and the PI3K superfamily, the activation loop (beginning with a Mg^2+^-interacting DFG motif) is important for both Mg^2+^/ATP and substrate binding. However, the ePK activation loops are longer than those in the PI3K superfamily, and in the ePKs the loop leads into a set of helices, starting with αF, that are not conserved in the PI3K superfamily (Kanev et al., 2019; Modi and Dunbrack, 2019). Instead, after the activation loop, the PI3K superfamily members have a set of helices unrelated to the ePKs, including helices kα9b and kα10. These helices partially overlap with a functionally important PIKK regulatory domain (PRD) also known as a negative regulatory domain (NRD) (Mordes et al., 2008). The catalytic subunit of PI3Kα, p110α, has a PRD-like region analogous to the PRD of PIKKs (Gong et al., 2023). The PRD in PIKKs is followed by a C-terminal helical region known as the FATC (residues 2518–2549 in mTOR), which folds back onto the kinase domain. In PI3Ks, the PRD-like region is followed by helices kα11 and kα12 (disordered in p110α), which are analogous to the FATC. However, instead of folding back onto the kinase domain, the kα12 is exposed and critical for membrane interaction (Chen et al., 2014; Hon et al., 2012; Miller et al., 2010; Zhang et al., 2011). Upstream of the kinase domain, the PIKKs have a conserved domain known as the FAT domain (named for the three proteins first characterized with this domain, mTOR/FRAP, ATM and TRRAP) (Bosotti et al., 2000), and the conserved PIKK FAT and kinase domains form a unit that is often collectively referred to as the FATKIN. Although not homologous with the FAT domain, the PI3Ks have a helical domain immediately N-terminal to the kinase domain that is structurally analogous to the C-terminal portion of the FAT domain (Fig. 2), and the helical/kinase unit in PI3Ks has been referred to as the HELCAT (Miller et al., 2010). All PIKKs have a large N-terminal helical solenoid region containing HEAT repeats preceding the FAT domain, with unrelated sequences among the PIKKs (Perry and Kleckner, 2003), and these N-terminal solenoids have structures specific to a given type of PIKK. The PIKKs and PI3Ks form large heteromeric complexes in which the PIKK or PI3K associates with regulatory subunits, and these regulatory interactions facilitate activation, inhibition, or localization of the enzymes. In their basal state, the PI3K superfamily enzymes have k_cat_ (catalytic turnover) values among the lowest of cellular enzymes (with k_cat_ < 0.1 s^−1^), however, when activated, their k_cat_s approach the mean for enzymes from the BRENDA database (about 10 s^−1^) (Davidi et al., 2018). We discuss here the structural mechanisms for this profound activation and the diverse ways these enzyme complexes achieve specific substrate recognition. For the PIKKs involved in DNA-damage response (DDR) and in the mTOR-containing complexes in mammals, the activation mechanisms involve similar features, and recent work with activated PI3Ks suggest that they too respond similarly. We focus on activating mechanisms for kinases including ATM, DNA-PK, mTORC1, PI3Kα, and VPS34 that have arisen from recent structural investigations.

## 2 Activation of PIKKs in DNA damage response

ATM, ATR, and DNA-PK are PIKKs that are activated in the DDR pathway, and every eukaryote has genes for at least one of these PIKKs (Blackford and Jackson, 2017). DNA-PK is activated by double-strand DNA breaks (DSBs) and is essential in repair by non-homologous end joining (NHEJ). ATM is also activated by DSBs and is needed for repair by homologous recombination. In response to various types of genotoxic stress, tracts of ssDNA coated with the ssDNA-binding replication protein A (RPA) complex, are formed, and these tracts recruit ATR (Zou and Elledge, 2003).

Ataxia telangiectasia (A-T) is a rare autosomal recessive disorder causing movement dysfunction (ataxia), cerebellar degeneration, immunodeficiency, radiation sensitivity and predisposition to cancer. The disease is associated with the absence of ATM kinase activity, due to mutations in the *ATM* gene. The kinase has a substrate specificity for a Ser or Thr followed by a Gln, and hundreds of ATM substrates have been proposed (Bensimon et al., 2010; Johnson et al., 2023; Kim et al., 1999; Matsuoka et al., 2007; Schlam-Babayov et al., 2021). ATM is activated in response to DSBs, enabling it to phosphorylate many targets involved in cell cycle checkpoints, DNA repair, apoptosis and senescence (Lee and Paull, 2021; Stuart and de Lange, 2024). It is also activated in response to oxidative stress (excess reactive oxygen species, ROS, production) (Guo et al., 2010b; Lee, 2024; Paull, 2015; Zhang et al., 2018), and oxidative stress associated with A-T contributes to its pathology. High-resolution structures have been reported for the basal state of human ATM (Baretic et al., 2017; Howes et al., 2023; Stakyte et al., 2021; Warren and Pavletich, 2022; Xiao et al., 2019) and a yeast ATM orthologue (TEL1) (Jansma et al., 2020; Wang et al., 2016; Xin et al., 2019; Yates et al., 2020). In response to double-strand DNA breaks (DSBs), ATM is activated by associating with a complex of MRE11/RAD50/NBS1 and DNA (Paull, 2015; Rotheneder et al., 2022), however, structural insight into this mechanism of activation is not yet available. An initial glimpse into the structural mechanisms of ATM activation was provided by low-resolution cryo-EM studies. One study reported both symmetric and asymmetric TEL1 dimers (Xin et al., 2019). The asymmetric dimer had one protomer with a basal-like conformation and a second protomer with a more compact conformation, in which the FAT and the kinase N-lobe were moved down toward the N-terminus, and the PRD was almost completely disordered. These changes were consistent with enlarging the substrate binding channel and suggested that the compact conformation was on the pathway to activation. In addition, a medium-resolution study of TEL1 reported structures of both symmetric, dimeric TEL1 and TEL1 monomers, with the monomers thought to represent the active enzyme having more open active sites (Xiao et al., 2019). These TEL1 monomers were lower resolution (7.8 Å), so details in the active site were limited, but they showed less ordered density in the PRD than was seen in the TEL1 dimers.

Growing evidence suggests that ATM has an important role in response to cellular oxidation and that loss of this function may be a primary driver for the cerebellar ataxia phenotype seen in A-T patients (Lee, 2024). Multiple disulfide linkages in the ATM were reported to form in response to redox stress (Guo et al., 2010b; Lee, 2024), but mutation of one of these cysteines in the PRD (C2991L) prevented redox signaling without affecting DNA-damage signaling (Guo et al., 2010b; Lee et al., 2018). In contrast to the C2991L, an R2579A/R2580A mutant in the FAT domain of ATM is not activated by double-strand DNA breaks but can be activated by redox stress. These two separation-of-function mutants (C2991L and R2579A/R2580A) have provided considerable insight into ATM-mediated signaling. ATM acts as a critical sensor of oxidative stress, and ATM deficiency is associated with accumulation of reactive oxygen species (ROS) (Lee, 2024; Lee et al., 2018; Lee and Paull, 2021; Zhang et al., 2018). A-T patients have greatly reduced levels of serum antioxidants (Reichenbach et al., 1999, 2002), and cells have reduced levels of vitamins A and E, decreased glutathione biosynthesis and lower levels of NADH and NADPH (Guo et al., 2010a).

**Fig. 3:**
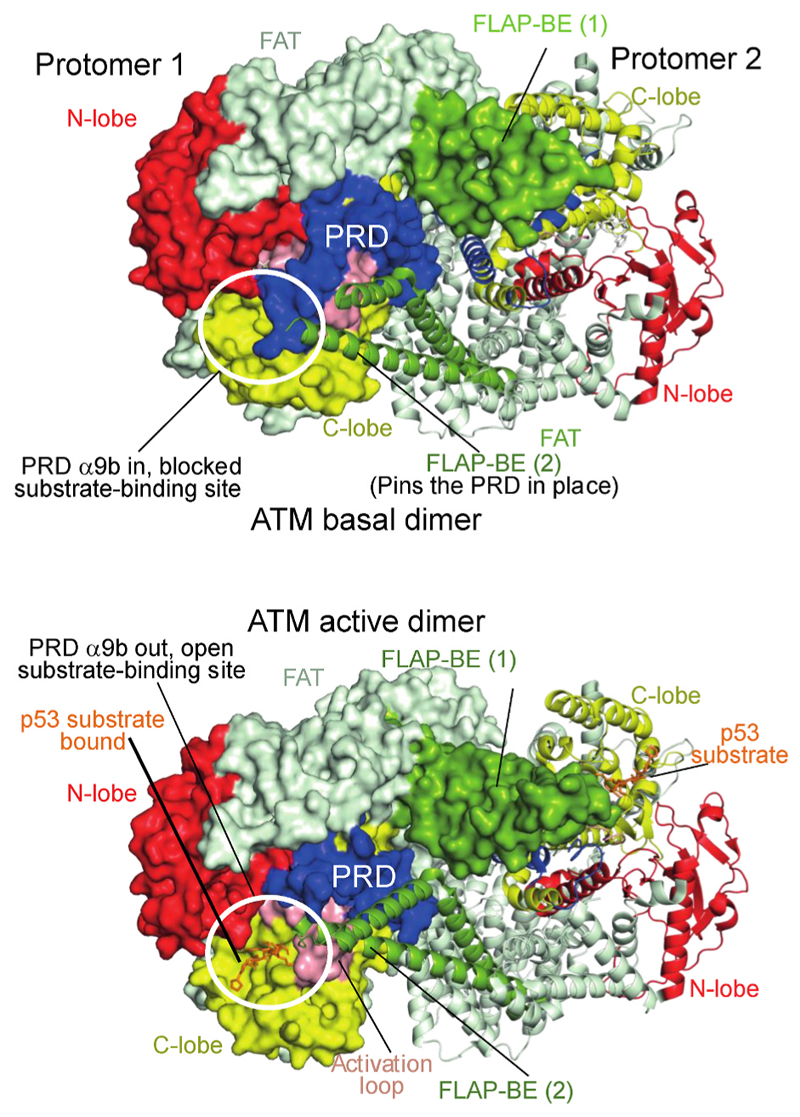
Activation of ATM kinase by oxidative stress twists the two protomers in the dimer relative to each other, thereby relieving PRD-mediated autoinhibition. The basal ATM dimer (upper, PDB ID 7SIC) has the PRD helix kα9b (residues 2954–3026, blue) blocking the substrate binding site and making pseudo-substrate interactions. In the basal state, the PRD is locked in place by the FLAP-BE (helices α21/α22, residues 2377 to 2476, green) in the FAT domain (light green) of the dimer-related protomer. Protomer 1 is illustrated in a surface representation, while protomer 2 is shown as ribbons. Upon oxidation, a disulfide bond forms between PRDs of the two protomers twisting the protomers relative to each other, accompanied by sliding of the N-lobe relative to the C-lobe (lower, PDB ID 8OXM). In the active dimer, the PRD becomes partially disordered. These changes expose the substrate site, allowing the p53 peptide substrate (orange) to bind to the optimally aligned active site.

**Fig. 4:**
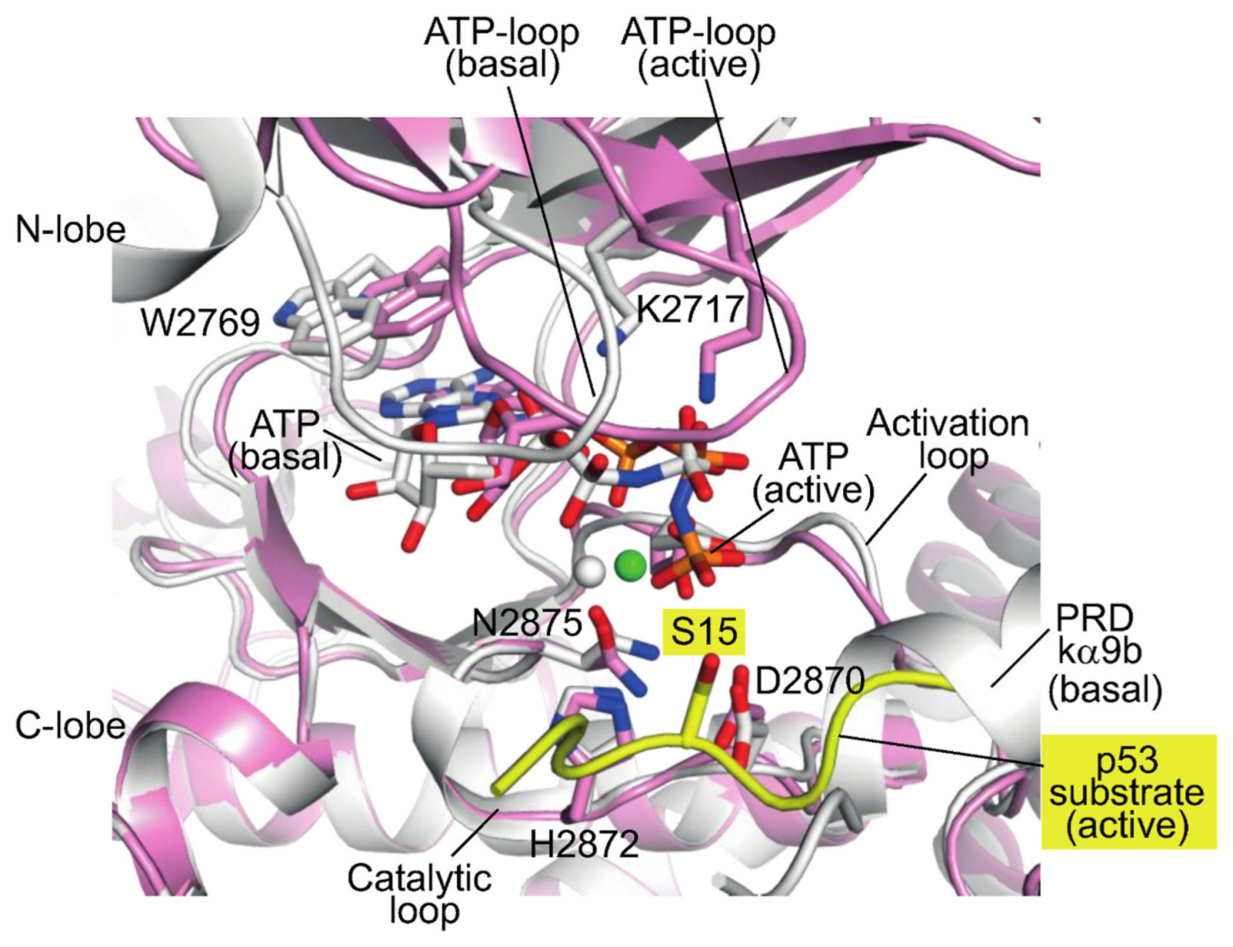
Oxidation-induced twisting of the ATM dimer results in realignment of the active site of each protomer. The kinase domain of REDOX-activated ATM (pink, PDB ID 8OXM (Howes et al., 2023)) is superimposed on the C-lobe of the basal state ATM (PDB ID 7SIC (Warren and Pavletich, 2022)). The N-lobe moves relative to the C-lobe, resulting in a substantial shift of the ATP-loop. The N-lobe movement brings Mg^2+^/ATP closer to the catalytic loop and the activation loop. The activation is accompanied by release of the PRD helix kα9b from the active site. The p53 substrate (yellow) was able to bind after release of the PRD, with the side chain of the substrate phosphoacceptor (Ser15 of p53 substrate) in contact with the γ-phosphate of the ATP.

Recently, a high-resolution structure has provided insight into the mechanism of activation of ATM by oxidative stress (Howes et al., 2023). In the basal conformation of the ATM dimer, the ordered portion of one protomer’s PRD (helix kα9b) is held in the peptide substrate-binding site by interactions with a helical hairpin known as the FLAP-BE from the other protomer in the dimer, and this blocks substrate binding (Fig. 3). In response to oxidation, C2991 from the PRD of one protomer in the ATM dimer forms a disulfide bond with C2991 from the other protomer (Guo et al., 2010b). Although the portion of the PRD containing C2991 is not ordered in the ATM structures, linking the two PRDs in the dimer together via a disulfide at this position would require the portion of the PRD following C2991 to be greatly strained if the dimer retained the conformation of the basal ATM dimer. To lessen this strain, one protomer rotates with respect to the other. This rotation releases the substrate-blocking PRD region, causes the FLAP-BE to rotate inward toward the dimer’s two-fold axis, and twists the kinase N-lobe relative to the C-lobe (Howes et al., 2023) (Fig. 3), causing a conformational change in the N-lobe that shifts the ATP-loop to bring the ATP γ-phosphate closer to the side chain hydroxyl of the substrate peptide (Fig. 4).

**Fig. 5:**
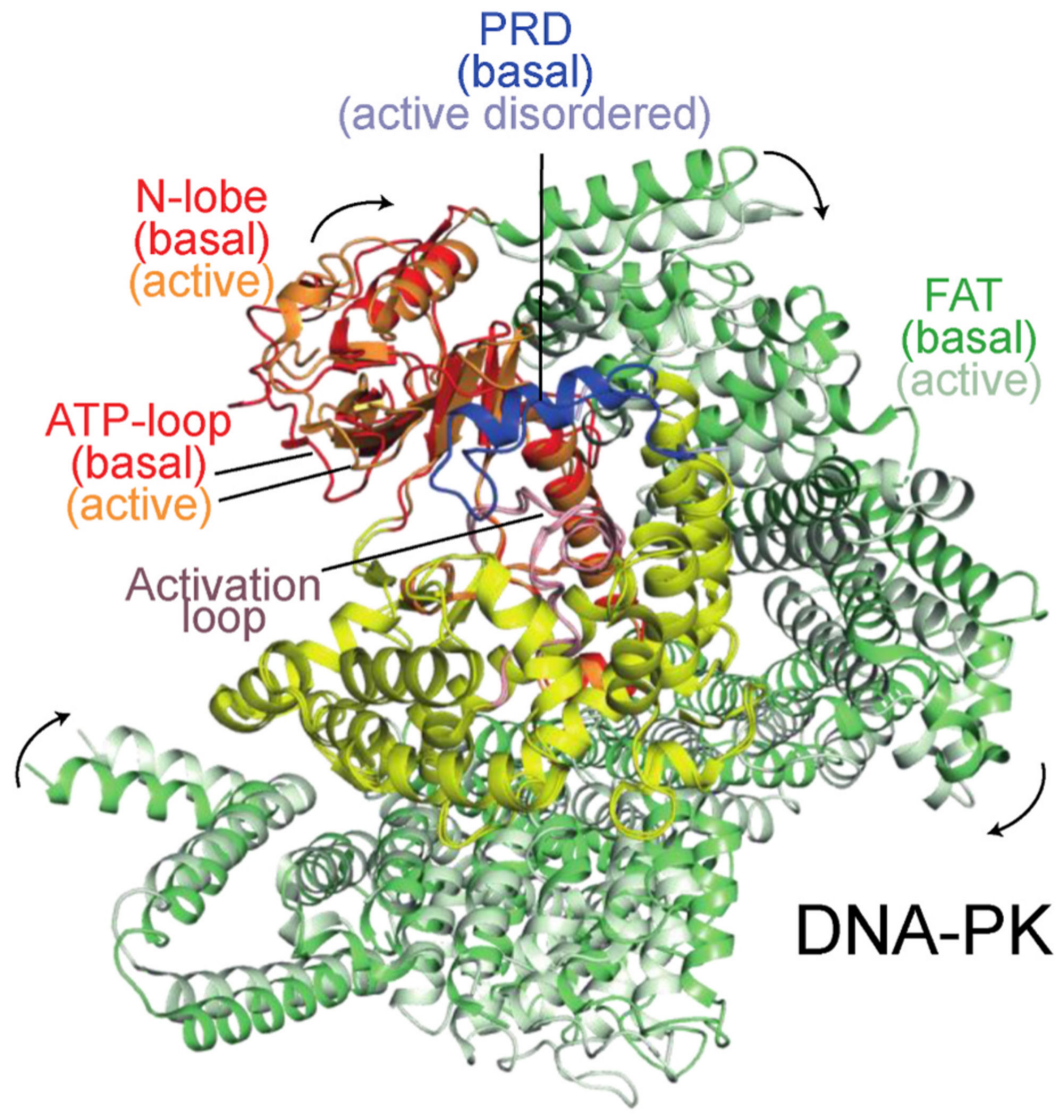
Upon activation, the N-lobe of DNA-PK is reconfigured to optimize interactions between ATP and the active site (PDB ID 7Z87) (Liang and Blundell, 2023). There is a shift in the ATP-loop of the DNA-PK catalytic subunit in a complex with Ku70/80, DNA, and a ligand (compound M3814) in the ATP-binding site. Activation results in the closure of the kinase domain around the ATP-site ligand. This ATP-loop shift resembles the shift resulting from RHEB binding to mTORC1 (PDB ID 6BCU). Only the kinase and FAT domains are shown. The basal and activated conformations are superimposed on the C-lobes of the kinase domain, and the activated conformation is colored with a lighter shade.

The redox stress-induced conformational changes in the ATM active site resemble changes caused by activating influences on other PIKKs: by RHEB binding to mTORC1 (Yang et al., 2017) and by an activating mutation for the yeast MEC1 (the orthologue of ATR) (Tannous et al., 2021). Inactive and active structures have also been reported for the DNA-PK holoenzyme. In a series of inactive DNA-PK complexes, the FAT and kinase unit (FATKIN) made only slight movements as a rigid body. However, in an ensemble of DNA-PK holoenzyme structures, one activated state was identified by the rotation of the PRD to open the substrate-binding groove for ATP and peptide substrates (Chen et al., 2021; Liang and Blundell, 2023). A more recent DNA-PK holoenzyme structure showed that ligand binding in the ATP site caused a further ATP-loop movement and full release of the PRD from the substrate binding site (Liang and Blundell, 2023). This open ATP/substrate groove resembles the active site of activated ATM (Howes et al., 2023). The active site changes in DNA-PK are linked to global conformational changes, which result in the FATKIN being pulled down towards the DNA end (Fig. 5). This movement is achieved through ordering of two structural elements that lay between the FATKIN and the DNA end (the N-terminal region of DNA-PKcs and an activation helix-hairpin-helix motif from the N-HEAT that binds to the DNA end) (Chen et al., 2021; Liang and Blundell, 2023).

## 3 Activation of lipid kinases PI3K*α* and VPS34

In mammalian cells, there are three classes of PI3Ks. Recent work has opened new doors to understanding the structures and activities of the class II PI3Ks (Kucukdisli et al., 2023; Lo et al., 2022, 2023) and the biology and molecular mechanisms of regulation of these enzymes has been reviewed recently (Koch et al., 2021). Here, we will focus on the class IA and class III PI3Ks.

### 3.1 Activation of PI3Kα

New insights into the dynamic regulation of the PI3Ks have arisen from development of small molecule activators of p110α, and from cryo-EM structures of complexes of PI3Kα activated by phosphorylated receptor mimics. The class IA enzymes have a p110 catalytic subunit (p110α, p110β, or p110δ) that associates with a p85-related regulatory subunit, and they can be activated upon association with bis-phosphorylated growth factor receptors. Structural features of these enzymes have been reviewed elsewhere (Burke, 2018; Rathinaswamy and Burke, 2020; Vadas et al., 2011; Vogt et al., 2023). Although the class I PI3Ks were the first enzymes of the PI3K superfamily whose structures were reported, initial structures of the class I enzymes captured the basal states of the enzymes (Berndt et al., 2010; Hon et al., 2012; Mandelker et al., 2009; Miller et al., 2010; Pacold et al., 2000; Walker et al., 1999; Zhang et al., 2011). Hydrogen/deuterium mass spectrometry (HDX/MS) suggested that activation of the class IA enzymes was accompanied by conformational changes and increased flexibility of p110/p85 complexes (Burke et al., 2011, 2012; Burke, 2018; Burke and Williams, 2013), but the full understanding of these changes generally awaited structural work by cryo-EM (Liu et al., 2021, 2022; Lo et al., 2022). Cryo-EM structures showed conformational variability of the p110α/p85α complex that was not seen in the crystal structures, with a population of the complexes having the activation loop in a DFG-out conformation, instead of the more usual catalysis-poised DFG-in conformation (Liu et al., 2021). Furthermore, in the activated state with a bis-phosphorylated peptide bound to the p85 regulatory subunit, the regulatory subunit is no longer visible in the structure. Although the regulatory subunit remained bound to the N-terminal adaptor binding domain (ABD) of p110α, it no longer interacted with the remainder of p110α subunit (Liu et al., 2021). This alternate, activated conformation arose due to disordering of ABD/RBD linker in p110α, as was suggested previously based on earlier HDX-MS analysis (Burke et al., 2012). The cryo-EM suggested that p110α/p85α exists in an ensemble of conformations, with characteristic global active and inactive populations.

**Fig. 6:**
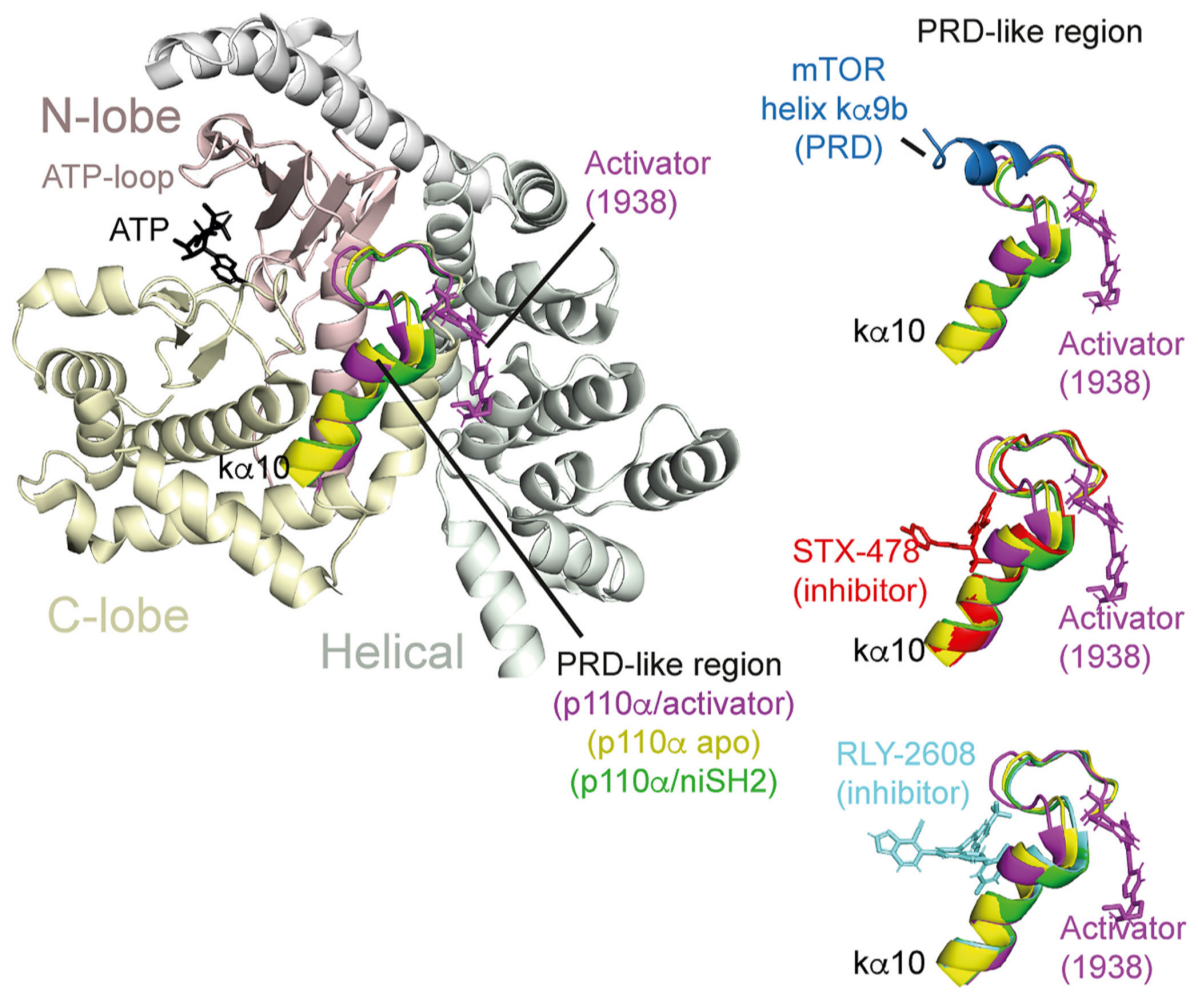
Allosteric activators and inhibitors of p110α bind on opposite sides of the PRD-like region. A ribbon diagram of the structure of a complex of human p110α bound to the small-molecule activator 1938 (magenta sticks) in an induced pocket, at the interface between the helical and kinase domains is shown on the left (PDB ID 8OW2). On the right, the helix kα10 and the preceding loop, which we refer to as the PRD-like region (residues 1009–1026), is shown in the same orientation for structures of the complexes of p110α with 1938, and with allosteric inhibitors STX-478 (red sticks, PDB ID 8TDU (Buckbinder et al., 2023),) and RLY-2608 (cyan sticks, PDB ID 8TSD (Varkaris et al., 2024),). In addition, the PRD-like region is shown for the p85-inhibited apo p110α/niSH2 complex (green, PDB ID 7PG5) and the apo p110α catalytic subunit with the ABD and kinase C-terminal membrane-binding helix kα12 deleted (yellow, PDB ID 8BFU). The helix kα9b from the mTOR PRD that restricts part of the active site of mTORC1 (blue, PDB ID 6BCX) is also shown superimposed on the PRD-like region from the p110α/1938 structure. All the structures were aligned on the C-lobe of the p110α/1938 complex (residues 851–1047).

**Fig. 7:**
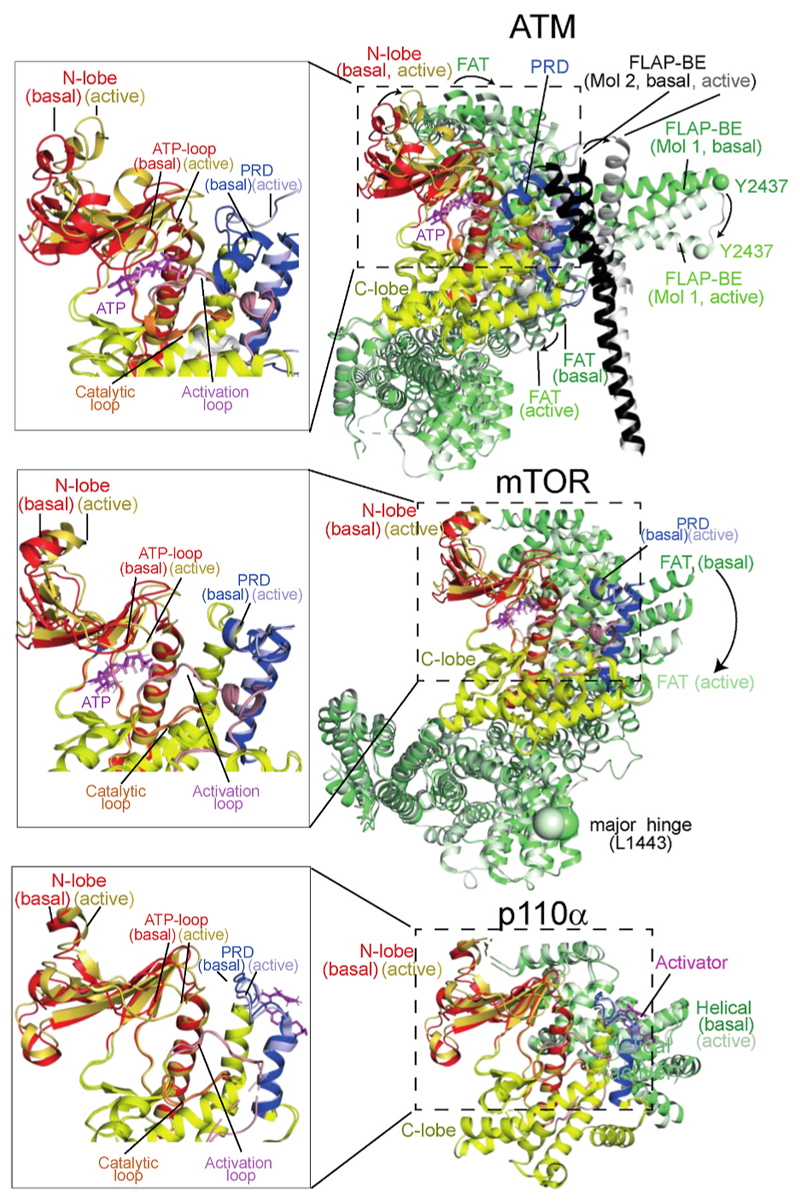
The helical and FAT domains of p110α and PIKKs shift upon activation to alter the relationship of the N-lobe of the kinase domain relative to the C-lobe. Upon activation, the FAT domains of the PIKKs ATM (upper, PDB IDs 7SIC for basal and 8OXM for H_2_O_2_-activated) and mTOR (middle, PDB ID 6BCX basal and 6BCU RHEB activated) slide along the kinase domain bringing the N-lobe closer to the C-lobe and shifting the ATP-loop to optimize phosphoryl transfer. A similar shift was seen for activator 1938 binding to p110α (bottom, PDB ID 8BFU for basal and PDB ID 8OW2 for 1938-activated). The basal and activated conformations are superimposed on the C-lobes of the kinase domains, and the activated conformation is colored with a lighter shade. For mTOR, the FRB was omitted for clarity.

Most recently, structures of p110α in the presence and absence of a small-molecule activator showed changes in conformation analogous to allosteric activation of mTORC1, ATM and DNA-PK (Gong et al., 2023). Part of the mechanism of activation for all these enzymes is a shift of the helical segment leading into the PI3K superfamily kinase domain, either the helical domain for PI3Ks or the FAT domain for PIKKs. For the members of the superfamily, this conformational shift can be caused by various influences: a small-molecule inducing a pocket at the helical/kinase interface for p110α (Figs. 6 and 7), an oxidation stabilized twisting of the dimer for ATM (Figs. 3 and 7), RHEB binding for mTORC1 (Fig. 7), and Ku/DNA/inhibitor binding for DNA-PK (Fig. 5). These results suggest that conserved features of the PI3K superfamily give rise to stereotypical changes in conformation upon activation, despite very different mechanisms transducing the activating event to the conformational response.

The activation of p110α by a small-molecule activator provided a unique window into the activation process (Gong et al., 2023) (Fig. 6). The activator induced a pocket at the kinase/helical interface of p110α, in a manner that was completely specific to this PI3K isotype. One wall of this induced pocket consists of the PRD-like region of the PI3Ks. The co-crystal structure of p110α with this small molecule activator, UCL-TRO-1938 (further referred to as 1938), suggested that one potential mechanism by which 1938 activates p110α is by shifting the PRD-like region away from the binding site of 1938 and towards the activation loop. In structures of p110α, the PRD-like region packs against the partially disordered activation loop, therefore this movement of the PRD-like region could promote the activation loop moving closer to the active site, enabling better positioning of a lipid headgroup and more efficient phosphoryl transfer. Remarkably, the other side of the PRD-like region forms a pocket into which allosteric, mutant-selective inhibitors of p110α bind (Gong and Vanhaesebroeck, 2024). Co-crystal structures of p110α with the allosteric inhibitors STX-478 (Buckbinder et al., 2023) and RLY-2608 (Varkaris et al., 2024) suggested that there is an allosteric network involving the C-terminal tail of the kinase domain, the DRH motif in the catalytic loop, and the activation loop. Molecular dynamics simulations suggested that the activating H1047R cancer-associated mutant is more likely than the wildtype enzyme to form a cryptic pocket that is capable of binding RLY-2608 or STX-478, which could account for mutant-favoring binding of these inhibitors (Kotzampasi et al., 2024). Comparison of structures bound to allosteric activators or allosteric inhibitors suggests that small molecules binding on different sides of the PRD-like region could cause different changes in the network of interactions involving the substrate-binding activation and catalytic loops, resulting in either inhibition or activation of enzyme activity. However, unlike 1938, binding of RLY-2608 and STX-478 did not cause movements in the PRD-like region. Interestingly, the amplitude of the movement of the PRD-like region may be correlated with the enzyme activity. The most activated complex (bound to 1938) had the greatest movement with respect to the p85-inhibited complex, and the free p110α catalytic subunit had a conformation of the PRD-like region that was intermediate between the 1938-activated and p85-inhibited ones (Fig. 6).

In addition to movement of the PRD-like region, 1938 also caused movement at the helical/kinase interface. Upon binding of 1938, the helical domain pivots, bringing the N-lobe of the kinase domain and the ATP binding ATP-loop closer to the ATP binding site (Fig. 7). This conformational shift of the N-lobe relative to the C-lobe is analogous to those observed upon mTORC1 activation by RHEB (Fig. 7). In a conformational change unique to p110α, 1938 induced movement of the C2 and helical domains relative to the C-lobe of the kinase domain (Gong et al., 2023), suggesting weakening of the inhibitory contacts at the p110α-helical/p85α-nSH2 and the p110α-C2/p85α-iSH2 domain interfaces.

Interestingly, the PRD-like region of PI3Kγ is also directly involved in both activation and inhibition of p110γ lipid kinase activity: the PRD-like region of p110γ (kα9/kα10 region) shows increased dynamics upon phosphorylation and activation by PKCβ, and decreased dynamics in the presence of a potent inhibitory, allosteric nanobody of PI3Kγ, although the phosphorylation site and the nanobody binding sites are distant from the catalytic site (Harris et al., 2023).

### 3.2 Activation of class III PI3Ks

**Fig. 8:**
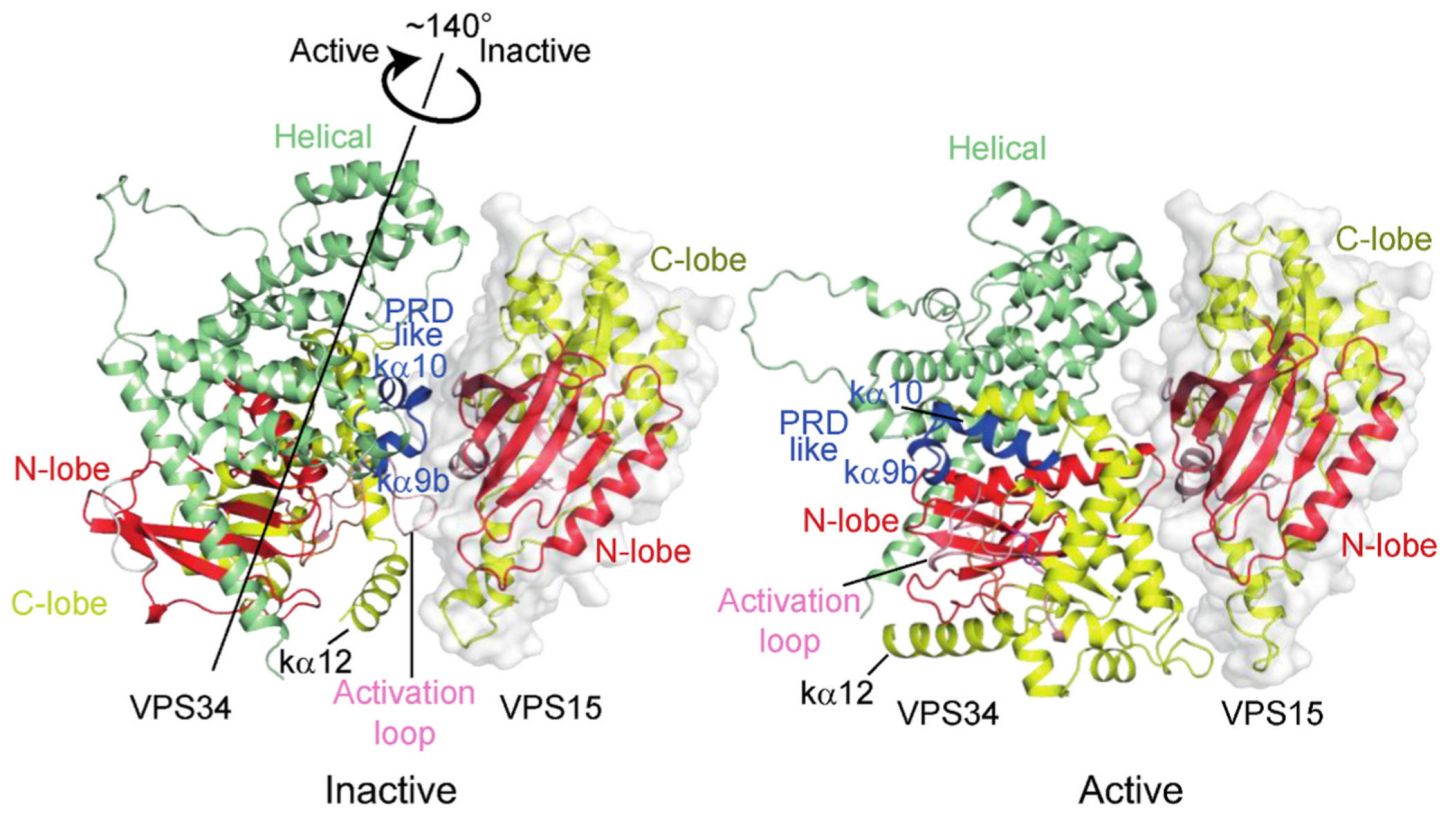
Inactive and active conformations of the VPS34 helical/kinase unit (HELCAT) relative to the VPS15 kinase domain. A recent cryo-EM study of the VPS34-C1 (Cook et al., 2023) showed two very different conformations of the Vps34 helical/kinase domain unit (HELCAT) that result in different contacts with the VPS15 kinase domain. These conformations were designated inactive and active. These two conformations are also predicted by AlphaFold 3. One predicted conformation (shown on right) is approximately the same as the active conformation observed by Cook et al., and it is predicted frequently by AlphaFold 3, but in a trial with 20 seeds, AlphaFold 3 also predicted the inactive conformation (left). A rotation of the Vps34 HELCAT from its position in the inactive form, by about 140° around the axis illustrated, brings the HELCAT to the active conformation. In the inactive conformation, the VPS15 kinase domain interacts with the VPS34 activation loop, while in the active conformation these interactions are broken, and the VPS34 activation loop points away from the VPS15 kinase domain.

The class III PI3K, VPS34, is likely most closely related to the progenitor of the PI3K superfamily, and it has an orthologue in all clades of eukaryotes, where it has critical roles in endocytosis, phagocytosis, and autophagy. VPS34 functions in two heterotetrameric complexes, each having unique functions: complex 1 (VPS34-C1, with VPS34/VPS15/BECLIN1/ATG14L subunits) initiates autophagy, while complex 2 (VPS34-C2, having the same subunits, except UVRAG replacing ATG14L) has a role in maturation of endosomes. The structures of these primordial PI3K complexes have been recently reviewed (Ohashi et al., 2019). The class III PI3K VPS34 complexes 1 and 2 (VPS34-C1 and VPS34-C2) have been studied by X-ray crystallography and cryo-EM (Baskaran et al., 2014; Cook et al., 2023; Ma et al., 2017; Rostislavleva et al., 2015; Tremel et al., 2021; Young et al., 2016, 2019). The crystal structure of yeast VPS34-C2 suggested an autoinhibited conformation, with the kinase domain of VPS34 closely associated with the N-terminal pseudokinase domain of the VPS15 subunit, sequestering the VPS34 activation loop and the C-terminal membrane binding helix kα12, two structural elements essential for VPS34 activation (Miller et al., 2010; Rostislavleva et al., 2015). In contrast to this tight association between the kinase domain of VPS34 and the pseudokinase domain of VPS15, several cryo-EM reconstructions of VPS34 complexes showed conformational classes in which the VPS34 helical/kinase domain (HELCAT) is dislodged from VPS15 and no longer visible in the structure (Baskaran et al., 2014; Chang et al., 2019; Cook et al., 2023; Young et al., 2019). Because there is no visible VPS34 HELCAT, the conformations of these complexes cannot be described as either active or inactive, and they have recently been referred to as transitional conformations (Cook et al., 2023). In attempts to capture the activated state of VPS34 complexes, structural analysis of VPS34 complexes bound to activators were carried out for the cryo-EM reconstruction of human VPS34-C1 complex bound to NRBF2 (Young et al., 2019) and a tomographic reconstruction of human VPS34-C2 bound to Rab5 on lipid vesicles (Tremel et al., 2021). Both complexes showed interaction between VPS15 and VPS34 kinase domains, but movement of the VPS34 HELCAT released the VPS34 activation loop from inhibitory constraints by VPS15, relative to the structure of yeast VPS34-C2, suggestive of an activated conformation of the complexes. The highest resolution reconstruction reported recently for VPS34-C1 bound to RAB1A, revealed one cryo-EM class with a catalytic arm similar to what was observed for the VPS34-C1/NRBF2 and VPS34-C2/Rab5/liposomes (Cook et al., 2023). The improved resolution enabled a more detailed view of VPS34 kinase domain interface with the VPS15 pseudokinase domain (PKD), revealing extensive interactions of the PRD-like region of VPS34 (kα9b and kα10, residues 829–840) with the N-lobe of the VPS15 PKD, as well as a contact of a residue in the VPS34 activation loop with the VPS15 N-terminus. This led to a proposal that all three of these structures (VPS34-C1 bound to RAB1A or to NRBF2 and VPS34-C2 bound to Rab5 on membranes) represent inactive states, or “on-pathway” states primed for further activation (Cook et al., 2023). Intriguingly, Cook et al. identified another cryo-EM class of VPS34-C1, although at lower resolution (about 5 Å for the VPS34 KD), in which the VPS34 HELCAT was ordered, but displayed a 140° rotation relative to the inactive conformation. This conformation, which was designated as the active conformation, has interactions between the VPS34 kinase domain and the VPS15 PK domain completely reconfigured, in a manner that would allow more extensive interactions of the complex with membrane and free access of the VPS34 catalytic site to the membrane-bound phosphatidylinositol substrate. The inactive and active conformations appear to be closely related to the two types of conformations shown in Fig. 8, as predicted by AlphaFold 3 (Abramson et al., 2024). Interestingly, a comparison of these two AlphaFold 3 predicted structures, suggested that the transition of the inactive to active state is accompanied by a small, but distinct realignment of the N-lobe of the kinase domain relative to the C-lobe, in the same direction (but with a lower magnitude) as observed for mTORC1 and ATM when they transition from the inactive to active form (Howes et al., 2023; Warren and Pavletich, 2022; Yang et al., 2017).

## 4 Modulation of mTOR in nutritional and growth factor signaling

mTOR is a Ser/Thr protein kinase that forms two types of complexes in eukaryotic cells, mTOR complex 1 (mTORC1) and mTOR complex 2 (mTORC2). The core of mTORC1 is a stable complex of three subunits: mTOR, LST8 and RAPTOR, while the mTORC2 core is a complex of four subunits: mTOR, LST8, RICTOR and SIN1. Both mTORC1 and mTORC2 form dimers with two-fold symmetry. Structures of mTOR-containing complexes and studies of reconstituted systems have provided insights into the mechanisms whereby these complexes recognize their substrates and are activated or inhibited. These have been summarized in recent reviews (Battaglioni et al., 2022; Cui et al., 2023a; Linde-Garelli and Rogala, 2023).

mTORC1 integrates signals from growth factors, amino acids, cholesterol and glucose to promote cell growth and proliferation when nutrients are sufficient, by activating anabolic processes such as protein, nucleotide and lipid synthesis and by inhibiting catabolic processes such as autophagy and lysosomal biogenesis (Goul et al., 2023; Liu and Sabatini, 2020; Napolitano et al., 2022; Szwed et al., 2021). mTORC1 is translocated to lysosomes through amino-acid dependent interactions with Rag GTPase heterodimers. This association with Rags is central to regulating mTORC1 activity in response to amino acids (Kim et al., 2008; Lama-Sherpa et al., 2023; Sancak et al., 2010). There are four Rag GTPases in mammalian cells, and they form heterodimers in which RagA or RagB binds to RagC or RagD. Rag heterodimers formed from GTP-loaded RagA or RagB and GDP-loaded RagC or RagD bind to the RAPTOR subunit of mTORC1, using their GTPase domains (Anandapadamanaban et al., 2019; Rogala et al., 2019). The Rags are, in turn, localized to lysosomes through interaction of their C-terminal roadblock domains with the lipidated Ragulator complex, a heteropentamer of LAMTOR1-5 subunits (Rogala et al., 2019; Su et al., 2017; Yonehara et al., 2017). The association of mTORC1 with the Rag/Ragulator complex is essential to regulation of mTORC1 by amino acid availability. On lysosomes, mTORC1 interacts with GTP-bound RHEB. The interaction of mTORC1 with RHEB allosterically activates mTORC1 (Yang et al., 2017). The complex TSC1/TSC2/TBC1D7 is a GTPase activating protein (GAP) for RHEB, which maintains RHEB in an inactive, GDP-bound state, incapable of interaction with mTORC1 (Dibble et al., 2012). Growth factor stimulated AKT phosphorylates TSC2 to inactivate its GAP activity leading to accumulation of GTP-bound RHEB and activation of mTORC1 (Ramlaul et al., 2021; Yang et al., 2021; Inoki et al., 2002; Manning et al., 2002; Menon et al., 2014). Recently, the C-terminal region of the ATP6AP1 subunit of lysosomal V-ATPase was shown to be an unconventional RHEB guanine nucleotide exchange factor (GEF) that can directly bind RHEB and stimulate its GTP loading, thereby activating mTORC1 on lysosomes (Feng et al., 2024). Nutrient facilitated recruitment of mTORC1 to lysosomes combined with RHEB-mediated activation downstream of growth factors enables mTORC1 to integrate these environmental cues.

## 5 Structural mechanisms of mTORC1 inhibition by DEPTOR

While RHEB is an allosteric activator of mTORC1, the protein DEPTOR binds to both mTORC1 and mTORC2 and was shown to be a partial inhibitor of mTORC1 (Heimhalt et al., 2021; Peterson et al., 2009; Walchli et al., 2021). DEPTOR consists of an N-terminal tandem DEP domain (DEPt) followed by an unstructured linker and a C-terminal PDZ domain. Recent structural studies provided insight into how DEPTOR inhibits mTORC1 (Heimhalt et al., 2021; Walchli et al., 2021; Weng et al., 2021). The PDZ domain binds tightly to the mTOR FAT domain, in a crevice between the FAT and the N-HEAT, however PDZ alone is not inhibitory, and instead PDZ acts as an anchor for DEPTOR’s inhibitory regions (Heimhalt et al., 2021; Walchli et al., 2021). The DEPt also binds to the mTOR FAT domain, but to a site different from the PDZ, and one model proposed that the DEPt binding prevents mTORC1 from adopting an activated conformation (Walchli et al., 2021). However, this model would not explain the observation that DEPTOR with its DEPt domain deleted is still able to inhibit mTORC1 (Heimhalt et al., 2021). At high concentrations, part of the DEPTOR unstructured linker binds to the FRB domain, which is a four-helix bundle (mTOR residues 2018–2114) insertion in the N-terminal region of the kinase domain, in a manner reminiscent of one type of substrate interaction (Heimhalt et al., 2021). The DEPTOR linker binding to the FRB was validated by NMR, HDX-MS and cryo-EM and a model was proposed that this binding to the FRB interferes with substrate binding in the active site and accounts for inhibition by both full-length DEPTOR and a construct lacking the DEPt domain (Heimhalt et al., 2021).

However, this would require a very stretched conformation of DEPTOR to be able to span from the PDZ to the FRB, and it would not then be possible for the DEPt domain to also bind to the FAT domain. A more recent report suggested a model that could explain observations from both DEPTOR/mTORC1 structures (Teh and Hisano, 2024), by taking into account the observation that DEPTOR can form dimers via domain-swapped DEPt domains. This model proposes that when DEPTOR binds to mTORC1 as a monomer, the DEPt is bound to the FAT domain, but not capable of inhibiting mTORC1 (because the protein is not long enough to reach from the DEPt binding site on FAT to the linker binding site on FRB and then to the PDZ binding site). However, when DEPTOR binds to mTORC1 as a dimer via domain-swapped DEPt domains, the DEPTOR dimer would be able to span from the PDZ binding site on the FAT to the linker-binding site on the FRB to block the kinase active site, and the DEPTOR linker would be available for phosphorylation as a substrate. In this model, the DEPt binding to the FAT domain would be a state that would not inhibit via competition at the FRB (Teh and Hisano, 2024), but it might allosterically prevent hyperactivation by RHEB or oncogenic mutation (Heimhalt et al., 2021; Walchli et al., 2021). Both monomers and DEPt-mediated dimers have been reported and modelled (Teh et al., 2020; Walchli et al., 2021; Weng et al., 2021), so it was proposed that the two cryo-EM reconstructions represent inhibitory DEPTOR dimers (Heimhalt et al., 2021) and non-inhibitory DEPTOR monomers (Walchli et al., 2021). However, this model of mTORC1 regulation by DEPTOR remains to be experimentally assessed.

## 6 Structural mechanisms of mTORC1 activation

Insights into the structural mechanisms of activation of mTORC1 were provided by high resolution cryo-EM structures of mTORC1 in its basal and RHEB-bound states (Yang et al., 2017). The FRB, LBE/LST8 and PRD helix kα9b form a deep cleft around the active site that limits substrate accessibility and negatively regulates mTOR. This restricted access prevents random encounter of substrate with the active site and enables regulation of phosphorylation by substrate recruitment.

An antibody that binds to a portion of the mTOR PRD region (also known as the negative-regulatory region, NRD) can activate mTOR and deletion of the antibody epitope, Δ2433-2451, activates mTOR (McMahon et al., 2002). This epitope corresponds to a partially ordered region of mTOR. The ordered part consists of PRD helix kα9b (2425–2436), which packs against the mTOR activation loop, closing off the end of the kinase active site, while the remainder of the epitope is contained in a disordered region (2438–2492) following kα9b (Fig. 6). Deletion of most of the unstructured region (2443–2486) did not affect activity, while deletion of part of helix kα9b increased the activity (Edinger and Thompson, 2004; McMahon et al., 2002; Sekulic et al., 2000; Yang et al., 2013), suggesting that helix kα9b plays an important role in restricting the active site (Fig. 6). A molecular dynamics study of an mTOR FATKIN showed that when the kα9b is in the active site, the active site exhibited only limited conformational variability and the PRD formed salt links with the FRB, bringing the FRB closer to the active site and further restricting substrate accessibility (Liu et al., 2024). However, the dynamics of a construct in which 2430–2450 in the PRD was deleted showed considerably greater variability, a more open catalytic cleft and more accessible secondary substrate-binding site on FRB.

The cryo-EM structure of full-length mTORC1 showed that the complex consists of two mTOR/RAPTOR/LST8 protomers associated with each other (Yang et al., 2017). The full-length mTOR consists of an N-HEAT region (17–932), an M-HEAT (933–1261), the FAT domain (1261–2001), and the kinase domain (2002–2549). When RHEB binds, parts of the N-HEAT, M-HEAT and FAT come together to form the RHEB-binding site. This requires a large conformational change in the N-HEAT and causes motions of the FAT domain at four hinges, with the major hinge motion of about 30° occurring at residue L1443 in the FAT domain. However, because the kinase C-lobe kα5/kα6 (2305–2315) and kα7/kα8 (2387–2395) loops pin the TRD1 region of the FAT domain (1364–1434) to the C-lobe, the hinging motion shifts the N-lobe of the kinase domain with respect to the C-lobe, thereby optimizing interactions in the active site for phosphoryl transfer (Fig. 7). Recently, the structure of a hyperactivating mTOR mutant associated with a rare pediatric kidney cancer was reported (Pacyna et al., 2024). This mutation is a duplication of 1455-EWED-1458 in the mTOR FAT domain, very close to the major hinge at L1443, that results in disordering of three helices at the site of the mutation. Surprisingly, although the activity of this mutant mTORC1 is at least as great as the activity of the RHEB-activated mTORC1, the cryo-EM structure of the mutant showed a global conformation that more closely resembles the apo mTORC1 than the RHEB-activated mTORC1. This suggests that the disorder at the site of the major hinge in mTOR caused by the mutation decreases the free energy of the transition state for the catalysis without changing the average conformational ensemble of the apo enzyme. This type of activation has been referred to as dynamic allostery (Cooper and Dryden, 1984; Huang et al., 2022), with the mutation presumably affecting the frequency or amplitudes of motions of the protein, without affecting the mean conformation. This suggests that the hinges might be targets for novel allosteric activators or inhibitors that could disorder a hinge or stabilize it, respectively. In addition to the disorder in the region of the mutation, the mTOR 1455-EWED-1458 duplication mutant caused an asymmetry of the complex, so that one of the RAPTOR molecules in the mTORC1 dimer was ordered, while the other had extensive disorder, suggesting that the activated mTORC1 mutant leads to loosening of interactions with the RAPTOR subunits.

## 7 Activation of mTORC2

mTORC2 phosphorylates members of the AGC kinase family: AKT, SGK, PKC, and PKN (Battaglioni et al., 2022). mTORC2 directly phosphorylates these AGC kinases in the TOR interaction motif (TIM, T443 in human AKT)(Baffi et al., 2021; Baffi and Newton, 2022) and the turn motif (TM, T450 in human AKT) (Oh et al., 2010). Another step in AGC kinase activation is phosphorylation of the hydrophobic motif (HM, S473 in human AKT1). HM phosphorylation is dependent on mTORC2 (Hresko and Mueckler, 2005; Sarbassov et al., 2005), and evidence has been presented for both direct phosphorylation by mTORC2, or indirect, by the AGC kinase itself once it has been activated by mTORC2 (Baffi et al., 2021; Baffi and Newton, 2022; Liu et al., 2014).

Based on studies in yeast, it has been suggested that the primordial role of TORC2 is regulation of plasma membrane homeostasis, and this role is preserved in metazoans (Riggi et al., 2020; Thorner, 2022). In addition, metazoan mTORC2 also has roles in signalling and cell metabolism. While many of these roles are upstream of mTORC1, others, such as plasma membrane homeostasis, cytoskeletal organization, cell survival, mitochondrial fitness, proliferation and migration have aspects that are independent of mTORC1. A key feature for most of these roles is that mTORC2 activates specific AGC kinases (reviewed in (An et al., 2021; Fu and Hall, 2020; Gaubitz et al., 2016; Liu and Sabatini, 2020; Ragupathi et al., 2024)).

Structures of mTORC2 and yeast TORC2 reported so far have investigated only the basal form of the enzyme, so it is not surprising that they are globally very similar with only minor local variations (Karuppasamy et al., 2017; Scaiola et al., 2020; Stuttfeld et al., 2018; Tafur et al., 2020; Yu et al., 2022). The mTORC1 activator RHEB does not activate mTORC2, and the RAPTOR subunit that plays roles in substrate recruitment and localization of mTORC1 is not a part of the complex. Although there are fewer studies showing what activates mTORC2, in comparison with what activates mTORC1, the mTORC2 field is rapidly expanding (An et al., 2021; Emmerstorfer-Augustin and Thorner, 2023; Fu and Hall, 2020; Ragupathi et al., 2024). An AKT/mTORC2 feedback loop, mTOR mutations, ribosomes, small GTPases, phosphoinositides, and membrane tension have all been shown to be activators of mTORC2, and we briefly summarize these below.

A positive feedback loop involving mTORC2 and AKT has been described, but mechanistic aspects are unknown (Yang et al., 2015). Furthermore, mTORC2 can be activated in vitro by some of the same mutations that activate mTORC1, for example, a four amino acid duplication mutant in the mTOR FAT domain that activates mTORC1 also activates mTORC2 in vitro (Pacyna et al., 2024). A genetic screen in yeast identified ribosomes as an activator of TORC2 (Zinzalla et al., 2011), and it was shown that active mTORC2 physically associates with ribosomes, that growth factor stimulation of PI3K increases this association, and that activation of mTORC2 by ribosomes is independent of protein synthesis. Ribosomes are involved in both the activation of mTORC2 and downstream substrate phosphorylation, since ribosome-associated mTORC2 is responsible for co-translational phosphorylation of the AKT turn motif (Oh et al., 2010). Only a subset of ribosomes activates mTORC2, and this activation may be restricted to regions where both mTORC2 and the ribosome are membrane associated (Zinzalla et al., 2011).

There are several indications that mTORC2 might be activated by association with Ras GTPases (reviewed in (Smith et al., 2020)). The SIN1 subunit has a RAS-binding domain (RBD), and crystal structures show that the RBD binds to K-RAS4A and H-RAS (Castel et al., 2021; Zheng et al., 2022). Although structural details of this interaction are clear, its impact on the mTORC2 activity is less clear. An RBD mutation preventing K-RAS4A interaction was reported to have no effect on mTORC2 function or assembly (Castel et al., 2021), however, optogenetic approaches showed that RAS locally activates mTORC2 in a growth factor signalling pathway to trigger actin reorganization, polarity, and directed migration (Lin et al., 2024; Pal et al., 2023). Furthermore, another report showed that the SIN1 PH domain inhibits the interaction of RAS with the RBD and that the RAS/SIN1 RBD interaction was necessary for full activation of SGK1 (Zheng et al., 2022). It is likely that new approaches will clarify the mechanistic details of the RAS/mTORC2 signaling axis.

mTORC2 associates with various cellular membranes, including plasma membrane, mitochondria, Golgi, endosomes, ER and mitochondria-associated ER membranes (Betz and Hall, 2013; Ebner et al., 2017). Membrane phosphoinositides, particularly PtdIns(3, 4,5)P3, have been reported to activate mTORC2, and a predominant view is that these lipids activate mTORC2 allosterically through the PH domain of SIN1, rather than by recruiting mTORC2 to membranes (Ebner et al., 2017; Gan et al., 2011; Liu et al., 2015; Schroder et al., 2007). Similarly, for the yeast Avo1 orthologue of SIN1, PtdIns(4,5)P2 is important for TORC2 activity, but not for membrane localization. Instead, the RICTOR orthologue Avo3 is the principal subunit enabling membrane localization (Emmerstorfer-Augustin and Thorner, 2023; Martinez Marshall et al., 2019).

Physical properties of the membrane can also activate or inhibit TORC2, and through a feed-back loop, TORC2 is an important regulator of plasma membrane homeostasis (reviewed recently (Fu and Hall, 2020; Ragupathi et al., 2024; Riggi et al., 2020; Thorner, 2022)). Membrane stress arising from inhibiting sphingolipid metabolism, hypotonic exposure, or mechanical stretch, activates TORC2 signalling. Membrane stretching in yeast causes redistribution of Slm1/2 proteins from membrane invaginations known as eisosomes, to punctate membrane compartments containing TORC2 (MCT) (Berchtold and Walther, 2009), triggering activation of TORC2 signalling (Berchtold et al., 2012; Niles et al., 2012). Recently, a cryo-EM reconstruction of near-native eisosomes revealed detailed interactions of individual PI(4,5)P2, PS and sterol molecules in the plasma membrane with the protein lattice covering the eisosome and showed that dynamic stretching of the protein lattice liberates the lipids (Kefauver et al., 2024). The elegant data support a model that the increased mobility of lipids in a stretched eisosome frees sequestered factors, such as Slm1/2 that are involved in TORC2 activation. The mechanistic details of Slm1/2-mediated TORC2 activation are largely unknown but may include Slm-dependent recruitment of Ypk1 (Ypk1 and Ypk2 are effector kinases of TORC2) to the plasma membrane for phosphorylation by TORC2 and Slm/calcineurin-dependent dephosphorylation of Avo2, which binds to TORC2 upon dephosphorylation, enabling optimal TORC2 activation (Berchtold et al., 2012; Leskoske et al., 2018; Niles et al., 2012). There are indications that mammalian mTORC2 is also regulated by membrane tension, based on the observations that mechanical stretching or membrane tension caused by hypo-osmotic shock result in phosphorylation of AKT S473 (Diz-Munoz et al., 2016; Kippenberger et al., 2005; Ono et al., 2022).

## 8 The PRD as a common regulatory feature in the PI3K-like superfamily

The importance of helix kα9b of the PRD in regulating mTOR is seen in other members of the PI3K superfamily enzymes. In SMG1 kinase, the large PRD domain (~1200 residues), negatively impacts SMG1 catalytic activity towards is substrate, UPF1 (Deniaud et al., 2015; Langer et al., 2020; Zhu et al., 2019). In apo ATM, helix kα9b acts as a pseudosubstrate, occupying the same site as p53 substrate until the redox-mediated activation dislodges it (Howes et al., 2023). Concomitant with helix kα9b vacating the substrate-binding site, the FAT domain slides relative to the kinase domain and twists the N-lobe of the kinase domain, optimizing the position of the ATP, bound to the N-lobe, for phosphoryl transfer (Fig. 7). For the PI3K p110α, the small-molecule activator 1938 binds on the surface of the PRD-like region (equivalent to mTOR kα9b/kα10). In this location, 1938 pries the p110α helical domain away from the PRD-like region, shifting the N-lobe of the kinase domain and causing the ATP-loop to take on a conformation like the RHEB-activated conformation of mTOR. In the class III PI3K, VPS34, the PRD-like region is a key element interacting with the inhibitory pseudokinase VPS15 (Cook et al., 2023; Rostislavleva et al., 2015).

## 9 Substrates make multiple interactions with PI3K superfamily members

It is common among the members of the PI3K superfamily that substrate interactions in the active site are modified by interactions with the kinase or kinase-associated subunits distant from the active site. This strategy is not surprising given that PI3K superfamily has its ancestral origin in a PI3K lipid kinase, and PI3Ks all make multiple interactions with their membrane substrates. For example, in the class I PI3Ks, the C2 domain, the iSH2 and kinase domains all interact with membranes (Burke, 2018; Gabelli et al., 2010; Rathinaswamy and Burke, 2020; Zhang et al., 2019). The class III PI3K complexes VPS34-C1 and VPS34-C2 both use Beclin1, VPS15 and VPS34 to interact with membranes (Chang et al., 2019; Cook et al., 2023; Fan et al., 2011; Huang et al., 2012; Ma et al., 2017; Noda et al., 2012; Ohashi et al., 2019, 2020, 2021; Rostislavleva et al., 2015; Stack et al., 1993; Tremel et al., 2021). The membrane binding elements in these subunits common to both complexes are hydrophobic features from the Beclin1 BARA domain (an aromatic finger (Huang et al., 2012; Noda et al., 2012), with contributions from two other hydrophobic loops (Ohashi et al., 2020), and a flipped out β-sheet (Chang et al., 2019)), the myristoylated N-terminus of VPS15 (Cook et al., 2023; Stack et al., 1993) and helix kα12 of VPS34 (Miller et al., 2010). The cryo-EM reconstruction of VPS34-C2 on an ER-mimicking lipid membrane bearing covalently attached RAB5A vividly showed that the primary interaction of the V-shaped complex with membranes was through the Beclin1 BARA domain positioned at the membrane surface (Tremel et al., 2021). In contrast, the catalytic arm was poised above the membrane, and it was proposed that this represents a semi-hopping mode of membrane encounter, with one arm of the complex making stable membrane interactions and the catalytic arm making only transient interactions. It may be that additional interactions with the membrane convert this semi-hopping mode to a scooting mode, where the complex stays associated with the membrane through multiple catalytic cycles. VPS34-C1 also binds membranes with its unique ATG14 subunit via an amphipathic helix known as the BATS domain that is important for localization of VPS34-C1 to membranes (Fan et al., 2011). Class I and III PI3Ks also augment their direct affinity for membranes by interacting with membrane-associated G-proteins (Buckles et al., 2020; Cook et al., 2023; Gillingham et al., 2019; Heitz et al., 2019; Rathinaswamy et al., 2021; Tremel et al., 2021). These accessory interactions of the PI3Ks with their membrane-containing substrates not only increase the localization to membranes, but they can also lead to allosteric activation or inhibition (Buckles et al., 2017, 2020).

Analogously, the mTOR complexes select their substrates, using one or more distinct substrate-binding sites (Battaglioni et al., 2022). In addition to substrate selection by the active site of the mTOR kinase domain, substrates are recruited by interactions with a range of binding sites on the mTORC1 surface: the TOS motif present in some substrates binds in a groove between the RNC and α-solenoid domain of RAPTOR (Yang et al., 2017), the RAIP motif binds to the RNC of RAPTOR (Bohm et al., 2021), an amphipathic helix present in many substrates binds to the FRB domain of mTOR (Yang et al., 2013, 2017), the DEPTOR PDZ and DEPt domains bind to separate surfaces of the mTOR FAT domain (Heimhalt et al., 2021; Walchli et al., 2021), a PRAS40 β-strand binds to the mLST8 WD40 domain (Yang et al., 2017), and TFEB binds to the mTORC1/Rag/Ragulator megacomplex, where TFEB interacts with both the RNC domain of the RAPTOR subunit and the RagA and RagC subunits of a non-canonically bound Ragulator/RagA/RagC complex (Cui et al., 2023b). For all these binding interactions, except for the binding in the active site of mTOR (the only common site for all mTORC1 substrates), there is at least one example providing structural insights into the binding mode, and a particularly elegant example is a study of the multisite hierarchical phosphorylation of 4E-BP1 (Bohm et al., 2021). Substrate binding by the mTORC2 complex uses the same phosphoryltransfer site on the mTOR subunit, but the RICTOR subunit prevents interaction with the FRB, and having no Raptor subunit, mTORC2 has no TOS-binding site. Instead, mTORC2 recruits substrates using the CRIM domain of SIN1, via an acidic loop that protrudes from the ubiquitin fold of the CRIM domain (Karuppasamy et al., 2017; Scaiola et al., 2020; Tatebe et al., 2017). Additional modes of substrate recruitment for mTORC2 are likely to exist.

## 10 Conclusions

The kinase domain in the PI3K superfamily has a conserved shape and flexibility. Key features of the kinase and associated domains, particularly the PRD and the FAT/helical region, enable the kinase domain to respond to activating influences through conformational changes. Considering recently discovered small-molecule activators of PI3Kα that exploit the flexibility of these regulatory elements, it may be possible to select for activators for all enzymes of the superfamily. Because activation typically involves coordinated conformational changes throughout the complexes, it is likely that there may be families of binding sites for such activators. These diverse activators could bind in various places yet promote the same conformational transition to an activated state.

## Data Availability

No data was used for the research described in the article.
